# Assessing the Need for Clinical Forensic Medicine Training in the Medical Undergraduate Curriculum

**DOI:** 10.7759/cureus.59545

**Published:** 2024-05-02

**Authors:** Manish Shrigiriwar, Harshal R Thube

**Affiliations:** 1 Forensic Medicine, All India Institute of Medical Sciences, Nagpur, IND

**Keywords:** skill-based teaching, casualty medical officer, medical graduate, hands-on training, clinical forensic medicine

## Abstract

Objective: This study was conducted to assess the need for clinical forensic medicine (CFM) training (hands-on and bedside) in medical undergraduate students and to determine the utility of skill-based teaching methodology in CFM.

Method: A cross-sectional study was carried out in the Government Medical College, where we used the mixed model approach (qualitative and quantitative component) to access the approach of three groups containing 50 participants each from (1) resident doctors/interns, (2) faculty, (3) casualty medical officers, toward skill-based teaching of CFM. A structured pretested questionnaire was administered to all study participants. The questionnaire was based on their perception regarding the legal problems faced during clinical practice and their attitude toward the need for CFM. It was followed by a focus group discussion (FGD) arranged separately for each group of 10 participants. Each FGD session lasts for 40 minutes with a moderator and recorder.

Result: Present MBBS (Bachelor of Medicine & Bachelor of Surgery) curriculum teaches CFM very early is strongly agreed by 20 (40%) of the faculty, four (8%) of interns, and three (6%) of medical officers. 40 (80%) of interns, 43 (86%) of medical officers, and 40 (80%) of faculty necessitate hands-on training in CFM in the MBBS curriculum. Forensic medicine specialists should handle clinical medico-legal cases (MLCs), as agreed by 50 (100%) faculty, 46 (98%) interns, and 47 (94%) medical officers.

Conclusion: Hands-on training in CFM is needed for a better practical approach for doctors toward handling MLCs. Hands-on training should be introduced in the second-year MBBS curriculum, and their clinical aspects should be taught simultaneously with clinical subjects till the internship.

## Introduction

Forensic medicine is now commonly used to describe all aspects of forensic work rather than just forensic pathology, which is the branch of medicine that investigates death. Forensic medicine is often used as an "umbrella terminology" to mean forensic pathology and CFM. Clinical forensic medicine (CFM) refers to the branch of medicine that involves an interaction among law, judiciary, and police officials, generally involving living persons. CFM is a term that has become widely used only in the last two or so decades. The practitioners of CFM have been given many different names throughout the years as police surgeons, forensic medical officers, forensic medical examiners, etc. Still, such names refer more to the appointed role than the work done. However, the term forensic physician has become more widely accepted nowadays [1].

CFM is assessing the physical condition of the living who allege that they are victims of an assault or examining the alleged perpetrator of the offense. It may cover various subjects, including forensic pharmacology, criminology, and traffic medicine [2]. CFM is the application of forensic medical techniques to living patients.

To address the unmet forensic needs of victims who are survivors of violent crimes and trauma, there is an urgent need to examine victims of violence by a specially trained person in medico-legal matters, i.e., clinical forensic physicians [3].

Currently, the role and scope of the specialty of CFM globally need to be defined, unlike other well-established medical specialties, such as gastroenterology or cardiology. In many cases, doctors practicing CFM or medical jurisprudence may only take on these functions as subspecialties within their general workload. Pediatricians, emergency medicine specialists, primary care physicians, psychiatrists, gynecologists, and genitourinary medicine specialists often have part-time roles as forensic physicians [4].

In developed countries, only doctors specialized in CFM or forensic pathologists can do examinations and prepare reports. Still, in India, even the duty or general medical officer with no knowledge of medico-legal cases (MLCs) is handed over to make such reporting [1].

Overall, the involvement of clinical toxicologists in CFM ensures that toxicological aspects of cases are properly addressed, leading to more accurate diagnoses, effective treatments, and reliable forensic investigations. Regulatory oversight and adherence to professional standards further contribute to the smooth functioning and credibility of the field. Medical education in India is guided by the regulations laid down by the Medical Council of India at the undergraduate and postgraduate levels [5]. Uniformly, the undergraduate level of teaching essentially consists of a curriculum spanning four-and-a-half years followed by 12 months of compulsory practical "on-hand" training (internship) leading to the award of a degree of medical graduation (MBBS (Bachelor of Medicine & Bachelor of Surgery)). There has been a considerable change in the curriculum of undergraduates, with early introduction to clinical settings is the other, which is being followed even in the West [6]. The students, early in their field, get to have first-hand exposure and knowledge of the various clinical skills as well as the subtleties of the doctor-patient relationships. Forensic medicine, as a part of the undergraduate curriculum, is taught in the second phase (third to fifth semester), along with the other three para-clinical subjects. The broad goal of teaching forensic medicine in our country is to produce a well-informed physician about the medico-legal responsibilities in medicine [6]. The objectives are that students would be capable of certain competencies like making accurate observations and inferring conclusions by logical deductions to aid in the administration of justice in all medico-legal problems; acquire the knowledge of the law in relation to medical practice including medical negligence and codes of medical ethics; ability to diagnose and manage common acute and chronic poisonings; and identify and adequately deal with the associated medico-legal problems.

There are seven days of compulsory and 15 days of optional training in casualty and forensic medicine, respectively, and is imparted during the internship wherein the student is expected to acquire the knowledge of various medico-legal responsibilities and learn to identify medico-legal problems, prepare medico-legal reports, conduct meticulous post-mortem examinations, diagnose and treat common poison conditions, etc., through supervised observation [7].

Despite the public interest and obvious shortcomings of medical practitioners in forensic medicine, these new graduates are facing serious problems in dealing with critical social issues such as domestic violence, child and adult sexual abuse, traffic medicine, and custodial medicine. They are directly posted to rural areas as medical officers in rural or primary health centers without supervision.

## Materials and methods

The objective of the study was to assess the need for casualty and forensic medicine training (hands-on, bedside) in second-year MBBS students, along with finding out the utility of skill-based teaching methodology in CFM.

Study setting

A present cross-sectional study was conducted in the Department of Forensic Medicine, Government Medical College, in the year 2021. Three groups were selected to participate in the study. A set of questionnaires tested each group. Group 1 is comprised of resident doctors/interns (n=50), group 2 contains faculty (n=50), and group 3 contains casualty medical officers/medical officers (n=50).

It is a mixed model approach study that includes both qualitative and quantitative components. Study participants were selected randomly as per their availability, and a structured pretested questionnaire was administered to all study participants physically. Those who had not given consent were excluded from the study. The questionnaire was based on their perception regarding the legal problems faced during clinical practice and their attitude toward the need for CFM. The questionnaires were validated by taking expert reviews from other forensic teachers. The wording, content, clarity, and response were corrected by taking review from fellow teachers. For responses, the Likert scale was used. Responses were noted on a scale from strongly agree, agree, neutral, disagree, and strongly disagree. This was followed by a focus group discussion (FGD) that was arranged separately for each group consisting of 10 participants. A fixed group discussion guide was prepared. Each session lasts for 40 minutes with a moderator and recorder. Different responses were recorded and then analyzed by the researcher. Responses were statistically analyzed in percentages. 

## Results

From the responses of faculties to the set questionnaire mentioned in Table 1 as percentages, it is observed that the majority of them (70%, n=35) agree that CFM is taught very early in the present medical curriculum. All of them emphasized on creation of a special unit for clinical medico-legal work in each hospital. Many (70%, n=35) believe hands-on training will improve the quality of medico-legal work. Most of them strongly agree (60%, n=30) that they hesitate while writing down their names at the end of the medico-legal report. A mixed opinion was there on the issue of informing every case of poisoning as an MLC. The majority of them (80%, n=40) agree that attempted suicide cases are MLC.

**Table 1:** Response of faculty members of medical college to questionnaires (%) MBBS, Bachelor of Medicine & Bachelor of Surgery; MLC, medico-legal case; MD, doctor of medicine

Questions	Strongly agree	Agree	Neutral	Disagree	Strongly disagree
1. Forensic medicine has relevance in clinical practice.	100	0	0	0	0
2. The CFM component taught in second-year MBBS is too early.	40	30	10	10	10
3. Interns are able to handle various MLCs referred to the hospital.	0	10	10	20	60
4. Medical officers can handle MLCs in your hospital efficiently.	0	0	20	70	10
5. At the end of the MBBS curriculum, students can issue the injury report efficiently.	0	0	0	30	70
6. Medical officer can assess the individual detained in police custody, who appears to have psychiatry disorder/mental health problem or learning disability.	0	0	0	20	80
7. Ideally MLCs should be handled by specialists in forensic medicine.	80	20	0	0	0
8. Do you think that there should be a specialized unit where victims of sexual assault are examined?	100	0	0	0	0
9. Specific qualification is required to deal with MLCs.	60	30	0	10	0
10. In an alleged H/o assault on police personnel, the medico-legal examination should be carried out.	50	50	0	0	0
11. MD (forensic) qualification is mandatory to practice CFM.	80	10	10	0	0
12. Do you think that medico-legal records should be maintained separately in your hospital?	60	20	10	10	0
13. Do you think CFM should be taught like regular clinical postings?	60	30	0	0	10
14. Are you independently capable of certifying the age of an individual?	40	50	0	0	10
15. While issuing the medico-legal report, doctors are afraid of putting their names.	60	40	0	0	0
16. Do you think CFM (hands-on training) will improve the quality of medico-legal certification?	70	10	0	20	0
17. In every case of poisoning it is mandatory to inform the police.	40	10	20	20	10
18. Casualty medical officer who handles MLC cases are experts in weapon examination.	0	0	0	60	40
19. Every alcoholic intoxication case is an MLC case.	10	10	0	50	30
20. Attempted suicide (rescue hanging) is an MLC.	30	50	10	10	0

Table 2 recorded the responses from an intern. As an intern before starting an actual medical practice, most (48%, n=24) agree that CFM has relevance in clinical practice. But they must be aware of the faculty (100%, n=50) in this opinion. 30% (n=15) of interns agree with the formation of a specialized unit of care for the victim of sexual assault, but 30% (n=15) disagree over this issue. Most (48%, n=24) disagree over mandatory reporting of each poisoning case to the police.

**Table 2:** Response of interns/resident doctors of medical college to questionnaires (%) MBBS, Bachelor of Medicine & Bachelor of Surgery; MLC, medico-legal case; MD, doctor of medicine

Questions	Strongly agree	Agree	Neutral	Disagree	Strongly disagree
1. Forensic medicine has relevance in clinical practice.	38	48	13	0	3
2. The CFM component taught in second-year MBBS is too early.	8	23	23	38	10
3. Interns are able to handle various MLCs referred to the hospital.	0	28	10	48	15
4. Medical officers can handle MLCs in your hospital efficiently.	0	18	18	53	13
5. At the end of the MBBS curriculum, students can issue the injury report efficiently.	10	23	20	40	8
6. Medical officer can assess the individual detained in police custody, who appears to have psychiatry disorder/mental health problem or learning disability.	70	25	3	3	0
7. Ideally MLCs should be handled by specialists in forensic medicine.	65	33	3	0	0
8. Do you think that there should be a specialized unit where victims of sexual assault are examined?	30	40	15	15	0
9. Specific qualification is required to deal with MLCs.	38	58	4	0	0
10. In an alleged H/o assault on police personnel, the medico-legal examination should be carried out.	50	43	5	3	0
11. MD (forensic) qualification is mandatory to practice CFM.	38	47	13	0	3
12. Do you think that medico-legal records should be maintained separately in your hospital?	10	60	20	5	5
13. Do you think CFM should be taught like regular clinical postings?	2	20	20	48	10
14. Are you independently capable of certifying the age of an individual?	42	38	10	10	0
15. While issuing the medico-legal report, doctors are afraid of putting their names.	40	50	8	2	0
16. Do you think CFM (hands-on training) will improve the quality of medico-legal certification?	35	45	3	17	0
17. In every case of poisoning it is mandatory to inform the police.	5	15	15	48	17
18. Casualty medical officer who handles MLC cases are experts in weapon examination.	10	20	28	35	7
19. Every alcoholic intoxication case is an MLC case.	35	55	3	5	2
20. Attempted suicide (rescue hanging) is an MLC.	30	50	10	10	0

A survey was taken of the medical officers who are the first point of contact for any medico-legal work and recorded in Table 3. 44% (n=22) of them do not think that teaching CFM is early in the second-year medical undergraduate curriculum. 50% (n=25) of them feel incapable of handling each MLC. The majority (62%, n= 31) disagree over the medico-legal assessment of mentally unstable persons in police custody single-handedly. 67% strongly agree that a forensic medicine person should handle MLC. All of them either strongly agree or agree over creating a specialized clinical medico-legal unit for the examination of victims of sexual assault at each hospital. The majority of them (44%) were not capable of certifying the age of an individual in an MLC. 

**Table 3:** Response of medical officers working in medical college to questionnaires (%) MBBS, Bachelor of Medicine & Bachelor of Surgery; MLC, medico-legal case; MD, doctor of medicine

Questions	Strongly agree	Agree	Neutral	Disagree	Strongly disagree
1. Forensic medicine has relevance in clinical practice.	66	34	0	0	0
2. The CFM component taught in second-year MBBS is too early.	6	22	16	44	12
3. Interns are able to handle various MLCs referred to the hospital.	12	0	22	60	6
4. Medical officers can handle MLCs in your hospital efficiently.	12	38	22	28	0
5. At the end of the MBBS curriculum, students can issue the injury report efficiently.	0	16	28	44	12
6. Medical officer can assess the individual detained in police custody, who appears to have psychiatry disorder/mental health problem or learning disability.	6	11	22	61	0
7. Ideally MLCs should be handled by specialists in forensic medicine.	64	12	12	6	6
8. Do you think that there should be a specialized unit where victims of sexual assault are examined?	72	28	0	0	0
9. Specific qualification is required to deal with MLCs.	22	62	10	6	0
10. In an alleged H/o assault on police personnel, the medico-legal examination should be carried out.	18	82	0	0	0
11. MD (forensic) qualification is mandatory to practice CFM.	44	50	6	0	0
12. Do you think that medico-legal records should be maintained separately in your hospital?	32	62	6	0	0
13. Do you think CFM should be taught like regular clinical postings?	12	72	6	0	10
14. Are you independently capable of certifying the age of an individual?	6	18	32	44	0
15. While issuing the medico-legal report, doctors are afraid of putting their names.	22	50	0	18	10
16. Do you think CFM (hands-on training) will improve the quality of medico-legal certification?	32	56	0	12	0
17. In every case of poisoning it is mandatory to inform the police.	61	33	6	0	0
18. Casualty medical officer who handles MLC cases are experts in weapon examination.	11	6	44	39	0
19. Every alcoholic intoxication case is an MLC case.	17	50	17	17	0
20. Attempted suicide (rescue hanging) is an MLC.	50	22	22	6	0

A fixed group discussion was started with participants on the topic of the current status of CFM. A total of 10 resident doctors, including interns, were interviewed for 40 minutes about different issues in handling MLCs in casualty. Starting with the basic concept of CFM, its purview and scope, present situations and difficulties they face in dealing with MLCs. After giving them a brief exposure to the basics of CFM, they gave different answers. Ranging from “We have been taught forensic medicine in 2nd year when we don’t even attend the clinics or be exposed to the patient” to “Most of part was related to post mortem but no ante-mortem.” One participant narrates, “As a resident doctor now, I feel I should have learned to subject with interest and injury report, age report, and certification should have been given hands-on experience.”

Similar views were taken from more experienced and clinically exposed medical professionals like teaching faculties. Their opinion is based on the teaching of students and their working pattern in the casualty or emergency department of medical college. “Most students are not exposed to the clinic during the second year, so they are not well oriented to see the clinical aspect of the subject.”

In the next session, casualty medical officers were interviewed about their experience at the first point of contact in any MLC. They needed to remember the theoretical part of dealing with MLCs but well versed in practical aspects. The response was, “I was posted directly after the internship as CMO, and when I started practising, I faced many MLCs I could not handle. I was apprehensive to report the things to the police. I then wondered why we were not taught this thing during the internship also.”

Many of them still face many problems in dealing with MLCs. The real concept is still not clear to them. The inclusion of CFM in the undergraduate curriculum and its impact on improving skills in handling MLCs are further discussed. The response from interns and resident doctors was affirmative for separate training in CFM. Their thinking is like removing fear and barriers while handling MLCs. One resident doctor said, “There was a patient who came with a lacerated wound on his hands, and I was unable to know whether it was self-inflicted or accidental. I rely on the patient saying it was accidental. I am not sure of it.” Another said, “Yeah same happened to me when I was confronted with a woman having multiple injuries on the body, and she told me it was an accidental one, so I didn’t report it to the police as I was not sure that it was a case of domestic violence.”

Victims of sexual assault are mainly dealt with in the obstetrics and gynecology department. A resident of obstetrics and gynecology (OBGY) said, “Mostly in examining rape victims, we don’t know how to do it, which injuries to see, what to preserve and how. We try to get over the cases somehow, but justice doesn’t follow.” Experienced faculty said that training would help clinicians to deal with MLCs properly. It will reduce the fear of legal medicine among clinicians and help improve justice. The surgeon said, “I fear if the cases with injury by knife or any heavy object come to me not for treatment but what to write in reports and how to face police investigations.” Many faculties have apprehension about attending the court after getting a summons for sexual assault cases, as they are not sure how court proceedings will go.

Many casualty medical officers were freshly appointed and were not oriented to sexual assault cases, and before joining as medical officers, they always had a fear of such cases in mind. They also have a fear of facing summons in the court of law, as many of them were not sure of their knowledge. So, if justifiable importance to clinical medico-legal training is given in the curriculum, they can handle the cases more effectively. One view from them was “In fear of inadequate knowledge, I don’t write my full name on injury certificates so that the court should not trace me.”

Overall all of them were of the opinion that it would improve the quality and timely justice and make them more confident in handling such cases. The discussion continued with the query about when and how the CFM should be included in the curriculum. All the resident doctors and interns said that it should be included in the curriculum with some weightage and taught in clinics at the time of posting. Some of them had a view of giving some extra hours of teaching to CFM.

All faculty said it should be included in the sixth or seventh semester, with clinical postings and exams at the end of the 7th semester. They had said, “Interns should be posted in casualty and exposed to such cases by forensic medicine experts.”

## Discussion

Danielson et al. conducted a similar kind of study, i.e., a mixed-methods study, including a web-based survey and FGDs, conducted among medical students who had completed their OBGY clerkship regarding the pelvic examination [8].

In our study, the opinions of various medical stakeholders regarding CFM were considered. The relevance of CFM was long back, as its origins go back many centuries. However, Smith rightly commented that “forensic medicine (cannot be thought of) as an entity until a stage of civilization is reached in which we have a recognizable legal system and an integrated body of medical knowledge and opinion” [9,10].

In-depth knowledge and practical hands-on experience in managing MLCs are very important. CFM always acts as a bridge of knowledge between medical and legal agencies in criminal investigations. The forensic physician who practices CFM usually presents the information orally to a court or other tribunal or forum in deciding the fate of the accused and victim [11].

Many times investigating agencies are in question, like in police custody mishaps. In that situation, CFM plays a vital role. Death-in-custody statistics are not always in the public domain; this remains the case, and investigating deaths in police custody may still not be independently undertaken [12]. Experience and up-to-date knowledge of recent laws, procedures, and protocols used for custody cases play a key role in successfully handling such cases. Instead of training in such protocol, many interns and casualty medical officers found it difficult to face such custody cases. This occurs probably due to early teaching of CFM and minimum exposure to actual cases in the compulsory internship. 

Another aspect of practicing clinical toxicology comes into the purview of CFM. Sharma et al. expressed their opinion that clinical toxicology and CFM should be practiced and not just taught in theory [13]. Forensic specialists should be in charge of all the MLCs reporting to casualty, and the admission and discharge of such cases should be informed to them on a priority basis. Every probable scenario of clinical toxicology cannot be taught in the theory lectures. This creates unnecessary delay and nonconfidence in clinical and medico-legal management of cases.

Need for clinical forensic medicine

The special drive should be taken as a program for the training of medical officers in CFM. This CFM program will address the unmet forensic needs of patients who are survivors of violent injury and trauma and those patients who have not yet succumbed to mortal injuries [14,15]. Currently, physicians and residents, principally from the specialties of emergency medicine, pediatrics, surgery, and gynecology, are performing clinical forensic examinations. While treating the patients the clinical doctor prioritizes the treatment, rather than focusing on the clinical forensic examination. These physicians generally have little forensic training and may be expected to render “expert forensic opinions” [16]. This creates unnecessary delay and under-quality medico-legal work, which will have a large percussion on future legal proceedings.

Common forensic errors of omission and commission occur with regularity in emergency departments [15,17]. From the above feedback, which has been received from faculties, interns/residents, and medical officers, it seems almost all people agree that forensic medicine has relevance in clinical practice following a study [1]. Most participants think that there should be a specialized unit to deal with sexual assault cases, and ideally, all MLCs should be handled by specialist forensic physicians. However, interns/residents and medical officers think that they can handle most MLCs efficiently but faculty in forensic medicine have differences in opinion.

Medical officers in casualty strongly disagree/disagree that medical officers are experts in weapon examination. Overall opinion of all participants who have given the feedback strongly agree/agree that hands-on training in CFM will improve the quality of medico-legal certification. Most feedback from the above-mentioned focus group thinks that medico-legal documentation should be maintained separately. Again, in our opinion, doctor of medicine (MD) forensic medicine/DFM qualification is mandatory to practice CFM. The teaching of the CFM component in the second-year MBBS is not too early, according to interns/residents and medical officers. Still, there is a difference in opinion from the faculty (FMT).

One more reason for below-standard medico-legal work by undergraduates is their ignorance and non-interest and unawareness toward CFM. All over the world, the absence of postgraduate training is often cited as the reason for graduates not entering forensic sciences or being unwilling to provide forensic services [18]. This study is following the other studies expressing that CFM should be introduced in the second-year MBBS curriculum. Clinical aspects should be taught simultaneously with clinical subjects till the internship.

Limitations of the study

The present study had a small sample size; it might not be representative of the entire population of medical students or educators. Also, the findings might not apply to all medical schools or regions, as the study was conducted in a specific geographical area or type of institution. The study was cross-sectional, so it might only capture a snapshot of opinions at a particular time, without accounting for changes in attitudes or needs over time. Factors other than the need for forensic medicine training might influence participants' responses, such as their prior exposure to forensic medicine or their career aspirations.

## Conclusions

The theoretical part of forensic medicine should start from the third semester till the sixth semester, along with clinical posting. Also, CFM and its application should be taught from the seventh semester of medical undergraduate teaching. The formative and summative assessment should be done after the completion of each semester for better evaluation. The internship in forensic medicine subject should not be optional and should be made compulsory for a minimum of 20 days. The study suggests the need for establishing a clinical forensic unit, and it is also strongly recommended by medical professionals.

## Appendices

1. Forensic medicine has relevance in clinical practice.

2. The CFM component taught in second-year MBBS is too early.

3. Interns are able to handle various MLCs referred to the hospital.

4. Medical officers can handle MLCs in your hospital efficiently. 

5. At the end of the MBBS curriculum, students can issue the injury report efficiently.

6. Medical officer can assess the individual detained in police custody, who appears to have psychiatry disorder/mental health problem or learning disability.

7. Ideally MLCs should be handled by specialists in forensic medicine.

8. Do you think that there should be a specialized unit where victims of sexual assault are examined?

9. Specific qualification is required to deal with MLCs.

10. In an alleged H/o assault on police personnel, the medico-legal examination should be carried out.

11. MD (forensic) qualification is mandatory to practice CFM.

12. Do you think that medico-legal records should be maintained separately in your hospital?

13. Do you think CFM should be taught like regular clinical postings?

14. Are you independently capable of certifying the age of an individual?

15. While issuing the medico-legal report, doctors are afraid of putting their names.

16. Do you think CFM (hands-on training) will improve the quality of medico-legal certification?

17. In every case of poisoning it is mandatory to inform the police.

18. Casualty medical officer who handles MLC cases are experts in weapon examination.

19. Every alcoholic intoxication case is an MLC case.

20. Attempted suicide (rescue hanging) is an MLC.
